# Usefulness of Machine Learning for Identification of Referable Diabetic Retinopathy in a Large-Scale Population-Based Study

**DOI:** 10.3389/fmed.2021.773881

**Published:** 2021-12-09

**Authors:** Cheng Yang, Qingyang Liu, Haike Guo, Min Zhang, Lixin Zhang, Guanrong Zhang, Jin Zeng, Zhongning Huang, Qianli Meng, Ying Cui

**Affiliations:** ^1^Department of Ophthalmology, Guangdong Provincial People's Hospital, Guangdong Eye Institute, Guangdong Academy of Medical Sciences, Guangzhou, China; ^2^Department of Ophthalmology, Dongguan People's Hospital, Dongguan, China; ^3^Shanghai Peace Eye Hospital, Shanghai, China; ^4^Xiamen Eye Center, Xiamen University, Xiamen, China; ^5^Department of Ophthalmology, Hengli Hospital, Dongguan, China; ^6^Information and Statistical Center, Guangdong Provincial People's Hospital, Guangdong Academy of Medical Sciences, Guangzhou, China

**Keywords:** diabetic retinopathy, machine learning, population-based study, classifier, XGBoost

## Abstract

**Purpose:** To development and validation of machine learning-based classifiers based on simple non-ocular metrics for detecting referable diabetic retinopathy (RDR) in a large-scale Chinese population–based survey.

**Methods:** The 1,418 patients with diabetes mellitus from 8,952 rural residents screened in the population-based Dongguan Eye Study were used for model development and validation. Eight algorithms [extreme gradient boosting (XGBoost), random forest, naïve Bayes, k-nearest neighbor (KNN), AdaBoost, Light GBM, artificial neural network (ANN), and logistic regression] were used for modeling to detect RDR in individuals with diabetes. The area under the receiver operating characteristic curve (AUC) and their 95% confidential interval (95% CI) were estimated using five-fold cross-validation as well as an 80:20 ratio of training and validation.

**Results:** The 10 most important features in machine learning models were duration of diabetes, HbA1c, systolic blood pressure, triglyceride, body mass index, serum creatine, age, educational level, duration of hypertension, and income level. Based on these top 10 variables, the XGBoost model achieved the best discriminative performance, with an AUC of 0.816 (95%CI: 0.812, 0.820). The AUCs for logistic regression, AdaBoost, naïve Bayes, and Random forest were 0.766 (95%CI: 0.756, 0.776), 0.754 (95%CI: 0.744, 0.764), 0.753 (95%CI: 0.743, 0.763), and 0.705 (95%CI: 0.697, 0.713), respectively.

**Conclusions:** A machine learning–based classifier that used 10 easily obtained non-ocular variables was able to effectively detect RDR patients. The importance scores of the variables provide insight to prevent the occurrence of RDR. Screening RDR with machine learning provides a useful complementary tool for clinical practice in resource-poor areas with limited ophthalmic infrastructure.

## Introduction

Diabetes mellitus affects 463 million adults and consumes 1.8% of gross domestic product globally, posing a huge burden on healthcare systems, especially in remote, underserved areas (1). Diabetic retinopathy (DR) is a vision-threatening condition that affects 22.27% of adults with diabetes (2). With the diabetes pandemic spreading from wealthy industrialized countries to developing regions, the number of people with DR will increase from 103.12 million in 2020 to 160.50 million in 2045 (2). Visual impairment and blindness due to DR can be significantly reduced if diagnosed at an early stage and treated appropriately. However, due to the high cost and low accessibility of eye services, <70% of people with diabetes receive eye examinations at regular intervals (3, 4).

The current strategy for detecting DR is based on clinical examination by an ophthalmologist or grading of retinal photographs via telemedicine, which relies on highly trained staff or requires expensive equipment. In addition, whether the recommended screening interval can be extended has attracted extensive debate because a large number of DR-negative patients receive repeated annual fundus screenings (5). It was estimated that the DR service would be reduced by 40% if people with no visible retinopathy at two consecutive screens received 2-year rather than annual screening in the Scottish Diabetic Retinopathy Screening programme (6). The National Health Service Foundation Trust claimed that screening people with type 2 diabetes every 2 years, rather than annually, would reduce screening costs by 25% (7). Therefore, establishing simple, practical methods for identifying people at high risk of referable DR (RDR) based on easily accessible indicators has become an important goal, which will help to target screening and prevention (8, 9).

Modeling for RDR is challenging because most medical data has a non-linear, non-normal, and non-independent distribution, and traditional regression analysis techniques would lose information (10). The use of machine learning (ML) techniques offers an alternative solution, which captures the non-linear relationship in data without prior assumption. Furthermore, ML is able to rank variables by importance. Previous studies have demonstrated that ML-based methods can accurately identify diabetes in the general population (11, 12). However, limited studies based on ML for DR classification are available to date (13, 14). To fill the knowledge gap, this study aims to develop RDR classifiers based on four ML techniques using simple non-ocular indicators and compare them with traditional logistic regression models to evaluate their usefulness in screening RDR in a large population-based survey.

## Methods

### Data Source and Participants

This study is a secondary analysis based on the Dongguan Eye Study (DES), which is a large-scale population-based survey conducted in Guangdong Province, Southern China (15, 16). The present study protocol was approved by the ethics committees of Guangdong Provincial People's Hospital. The study was performed in accordance with the Declaration of Helsinki. Written consent was obtained from all participants before entering the study.

The detailed methodology of the DES has been reported in previous articles (15, 16). In brief, 11,357 eligible participants residing in Hengli Town, Dongguan City were recruited between September 2011 and February 2012, with 8,952 (response rate 78.8%) completing the systemic and ophthalmic examinations. Standardized questionnaires were used to obtain data on demographics, lifestyle, socio-economic status, quality of life, and medical and family history. Height, weight, waist and hip circumference, and blood pressure were measured using standard protocols. Fasting venous blood was collected to obtain the following measurements: fasting blood glucose (FBG), hemoglobin A1c (HbA1c), total cholesterol (TC), triglycerides (TG), high-density lipoprotein cholesterol (HDL-C), low-density lipoprotein cholesterol (LDL-C), and blood uric acid (UA). All participants with diabetes or hypertension received a comprehensive ocular examination that covered visual acuity, automatic refraction, slit lamp, intraocular pressure, and retinal photography.

### Definition of the Outcome

The diagnosis of diabetes is based on medical history and endocrinologists' records, the use of insulin therapy, oral hypoglycaemic drugs, or the latest criteria according to the Chinese Guidelines for the Management of Diabetes Mellitus in the Elderly (2021 Edition): typical symptoms of diabetes mellitus (irritable and excessive drinking, polyuria, polyphagia, and unexplained weight loss) plus random plasma glucose ≥ 11.1 mmol/L; FBG ≥ 7.0 mmol/L; 2-h plasma glucose level ≥ 11.1 mmol/L during a 75-g oral glucose tolerance test (OGTT); or HbA1c ≥ 6.5%. Re-testing on another day was performed to confirm the diagnosis.

The DR status was graded based on fundus photography according to the classification system designed by the Wisconsin Epidemiologic Study of Diabetic Retinopathy (WESDR) and the Early Treatment Diabetic Retinopathy Study (ETDRS) (17). Diabetic macular oedema (DME) was diagnosed according to the International Diabetic Macular Edema Severity Scale, defined as having significant retinal thickening or hard exudate in the posterior pole. Fundus fluorescein angiography (FFA) was performed to confirm the diagnosis in participants with suspected severe non-proliferative diabetic retinopathy (NPDR) or proliferative diabetic retinopathy (PDR), macular edema, retinal vasculopathy, posterior uveitis, or other retinochoroidal diseases. DR was categorized into no diabetic retinopathy, mild NPDR, moderate NPDR, severe NPDR, or PDR. RDR was adopted as the primary outcome in the present study, which was defined as the presence of moderate NPDR, severe NPDR, PDR, or DME. RDR is clinically essential because RDR people will be referred to ophthalmologists for review in a diabetic retinopathy screening programme, while those without RDR will continue to be screened in primary care (18).

### Variables for Modeling

Table 1 shows the potential variables included in the model, including age, gender, body mass index (BMI), waist-to-hip ratio (WHR), waist circumference, blood pressure, lifestyle, and medical and family history. Lifestyle information included smoking, alcohol consumption, and dietary habits. Those who smoked at least one cigarette a day for 6 months were defined as smokers, while those who drank alcohol at least once a week for 6 months were defined as drinkers. Duration of diabetes was defined as the time between the first diagnosis of diabetes by an endocrinologist and entry into this study. For newly diagnosed diabetes, diabetes duration was defined as 0 years. In addition, laboratory serum parameters were included, as these tests are routine performed in government health institutions in China.

**Table 1:** A list of the variables that were used for modeling in this study.

**Demographics and anthropometry**
**Age, sex, marital status, number of children, occupation, education level, economic status/income, height, weight, body mass index, waist circumference, hip circumference, waist/hip ratio, systolic blood pressure, diastolic blood pressure**.
**Disease history and comorbidity**
**Duration of diabetes, duration of hypertension, duration of hyperlipidemia, history of cardiovascular disease (CVD), anti-hypertension drugs, use of insulin, family history of diabetes, family history of hypertension, family history of diabetes**.
**Lifestyle and habitus**
**Smoke status, years of smoking, smoking amount, alcohol use, years of drinking, drinking amount**
**Biochemistry parameter**
**Fasting glucose, HbA1c, total cholesterol (TC), triglycerides (TG), high-density lipoprotein cholesterol (HDL-c), low-density lipoprotein cholesterol (LDL-c), serum creatinine (SCr), uric acid (UA), blood urea nitrogen (BUN), endogenous creatinine clearance rate (CCr), microalbuminuria (MAU)**

### Statistical Analysis

All analyses were performed in R software version 4.0.3. The distribution of demographic and clinical characteristics was presented using mean ± standard deviation (SD) for continuous variables and by number and percentage for categorical data. Differences between RDR and non-RDR patients were evaluated by using the independent *t*-test for continuous normally distributed variables, the Mann-Whitney test for non-normally distributed variables, and the chi-squared test for categorical variables. All tests were two-tailed, and *p* < 0.05 was considered to be statistically significant.

**Figure 1:**
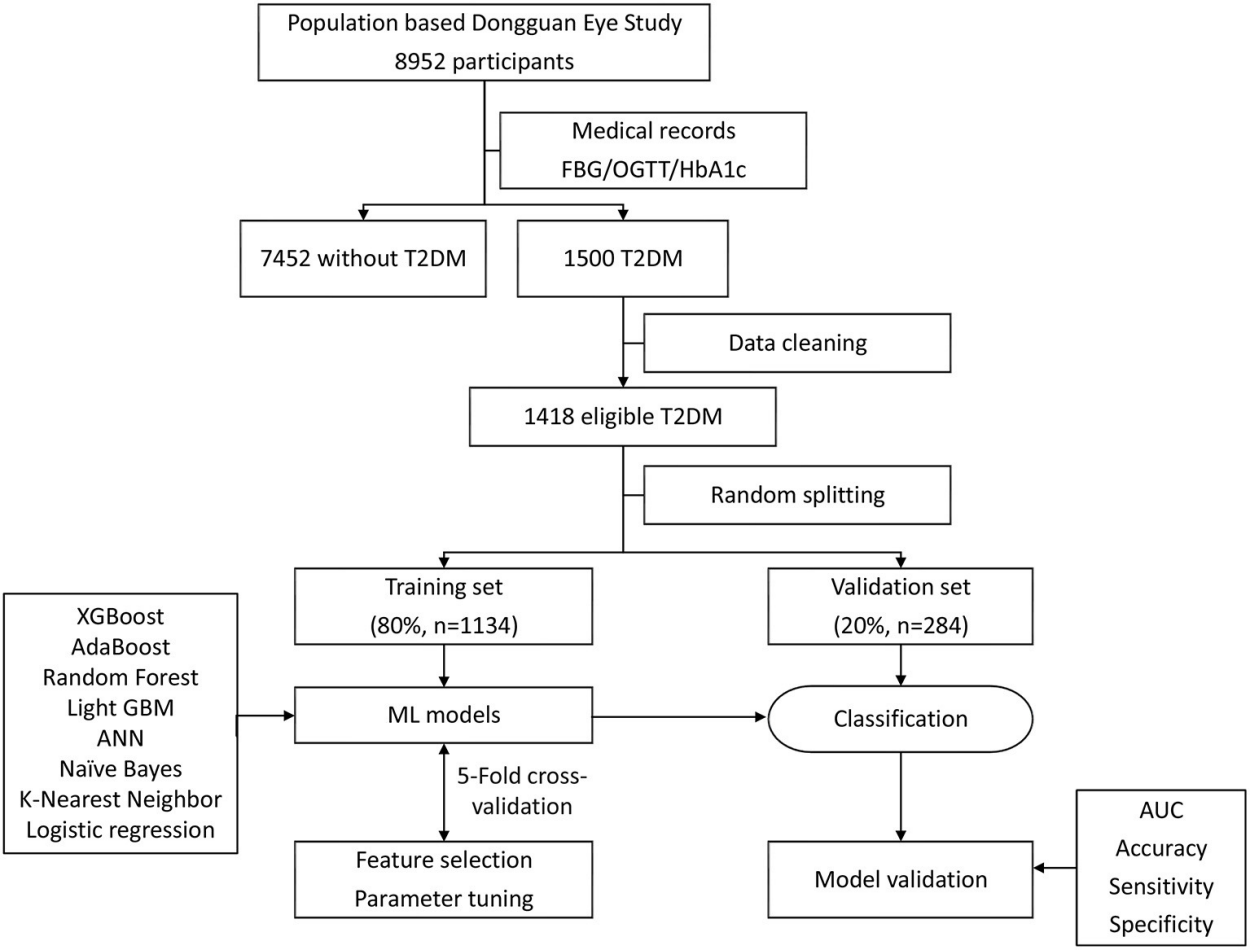
Machine learning flowchart of this study. ML, machine learning; XGBoost, extreme gradient boosting; ANN, artificial neural network; AdaBoost, adaptive boosting; GBM, gradient boosting machine.

Figure 1 shows the analytical framework for this study. Eight algorithms were used to construct models for detecting RDRs: extreme gradient boosting (XGBoost), random forest, naïve Bayes, k-nearest neighbor (KNN), AdaBoost, Light gradient boosting machine (GBM), artificial neural network (ANN), and logistic regression. ML techniques were able to calculate the importance of the variables, i.e., the effect of each variable on the generated model of statistical significance. To identify the most important features for diagnosing RDR (Table 1), we applied XGBoost, random forest, naïve Bayes, and KNN to rank the importance of the variables. The top 10 variables that were present in all four ML algorithms were entered into the subsequent model development. After data cleaning, the data were randomly divided into a training set and a test set (at an 80:20 ratio) to assess the reliability of these classifiers. To obtain the realistic and generalisable estimates as well as conservative confidence intervals, five-fold cross-validation and variance estimation were performed. Each model was fitted based on the training dataset, and its accuracy was assessed on the test dataset. The area under the curve (AUC) of receiver operating characteristic (ROC) curves was calculated to evaluate the performance of each model.

## Results

Table 2 summarizes the demographic and clinical characteristics of the included participants. A total of 1,418 eligible patients with diabetes (82 RDR and 1,336 non-RDR) were included in the model development and internal validation. The mean ages of participants with RDR and non-RDR were 61.1 ± 10.7 years and 60.0 ± 11.0 years, respectively. The participants with RDR had higher systolic blood pressure (SBP), a longer duration of diabetes, higher levels of FBG and HbA1c, and a higher proportion of family history of diabetes (all *p* < 0.05). Other characteristics were similar in the two groups, such as sex, BMI, WHR, and BUN (all *p* > 0.05).

**Table 2:** Characteristics of the included participants.

	**Without RDR**	**With RDR**	***P*** **-value**
No. of subjects	1,336	82	
Age, year	60.0 (11.0)	61.1 (10.7)	0.389
Male, *n* (%)	572 (42.81)	42 (51.22)	0.136
Current smoker, *n* (%)	350 (26.20)	21 (25.61)	0.906
Body mass index, kg/m^2^	26.35 (4.19)	25.90 (3.51)	0.367
Waist-to-hip ratio	0.91 (0.07)	0.91 (0.06)	0.355
Systolic blood pressure, mmHg	141.7 (20.1)	147.1 (19.8)	**0.023**
Diastolic blood pressure, mmHg	78.8 (11.2)	79.1 (10.9)	0.787
Duration of diabetes, year	1.33 (2.82)	5.1 (5.5)	**<0.001**
Fasting blood glucose, mmol/L	7.80 (5.22)	9.54 (4.48)	**0.002**
HbA1c, %	7.01 (1.67)	8.03 (2.05)	**<0.001**
Blood urea nitrogen, mmol/L	6.44 (14.81)	6.39 (2.59)	0.979
Serum creatine, μmol/L	78.13 (43.93)	83.61 (30.32)	0.307
Triglyceride, mmol/L	2.10 (2.12)	2.13 (2.70)	0.919
Total cholesterol, mmol/L	5.45 (1.26)	5.59 (1.32)	0.374
Use of insulin, *n* (%)	11 (0.82)	2 (2.44)	0.136
Anti-hypertension medication, *n* (%)	354 (26.50)	25 (30.49)	0.428
History of hypertension, *n* (%)	477 (35.70)	35 (42.68)	0.202
History of hyperlipidemia, *n* (%)	162 (12.13)	12 (14.63)	0.502
Family history of hypertension, *n* (%)	364 (27.25)	24 (29.27)	0.690
Family history of diabetes, *n* (%)	165 (12.35)	20 (24.39)	**0.002**

*RDR, referable diabetic retinopathy. The bold values indicate statistically significant*.

### Relative Importance of Variables

**Figure 2:**
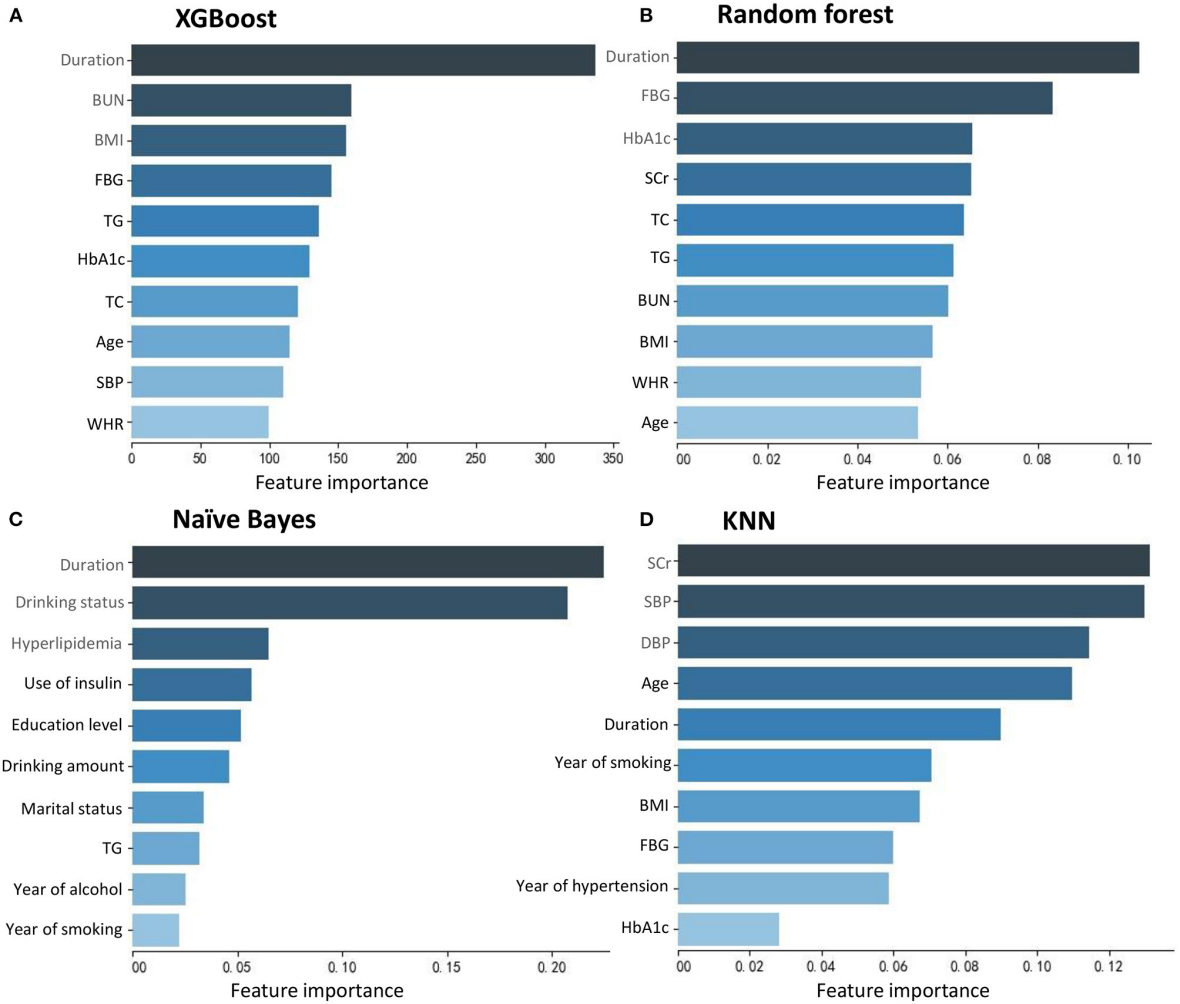
Feature importance contributed to each machine learning model. **(A)** XGBoost. **(B)** Random forest. **(C)** Naïve Bayes. **(D)** KNN.

**Figure 3:**
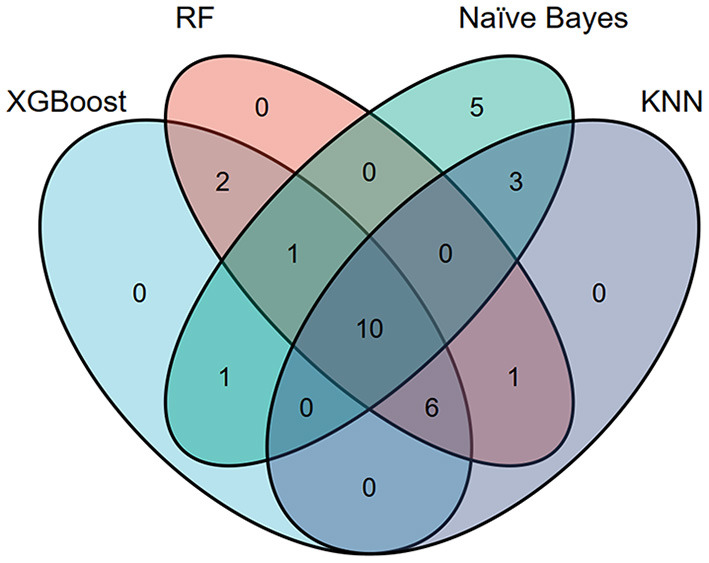
Venn plot showing the most important features in each model for detecting referable diabetic retinopathy.

Figure 2 shows the top 10 features for RDR in the four ML algorithms. The duration of diabetes, BUN, BMI, FBG, TG, HbA1c, TC, age, SBP, and WHR were identified as the 10 most important factors in the XGBoost model. The duration of diabetes, FBG, HbA1c, SCr, TC, TG, BUN, BMI, WHR, and age ranked as the 10 most important factors in the random forest model. In the naïve Bayes model, the top 10 factors were the duration of diabetes, drinking status, history of hyperlipidaemia, use of insulin, education level, daily alcohol consumption, marital status, TG, duration of drinking, and duration of smoking. Among the top 20 factors in each model, there were 10 essential factors present in all models: the duration of diabetes, HbA1c, SBP, TG, BMI, SCr, age, education level, duration of hypertension, and income level (Supplementary Table 1; Figure 3).

### Performance of the ML Algorithms

**Figure 4:**
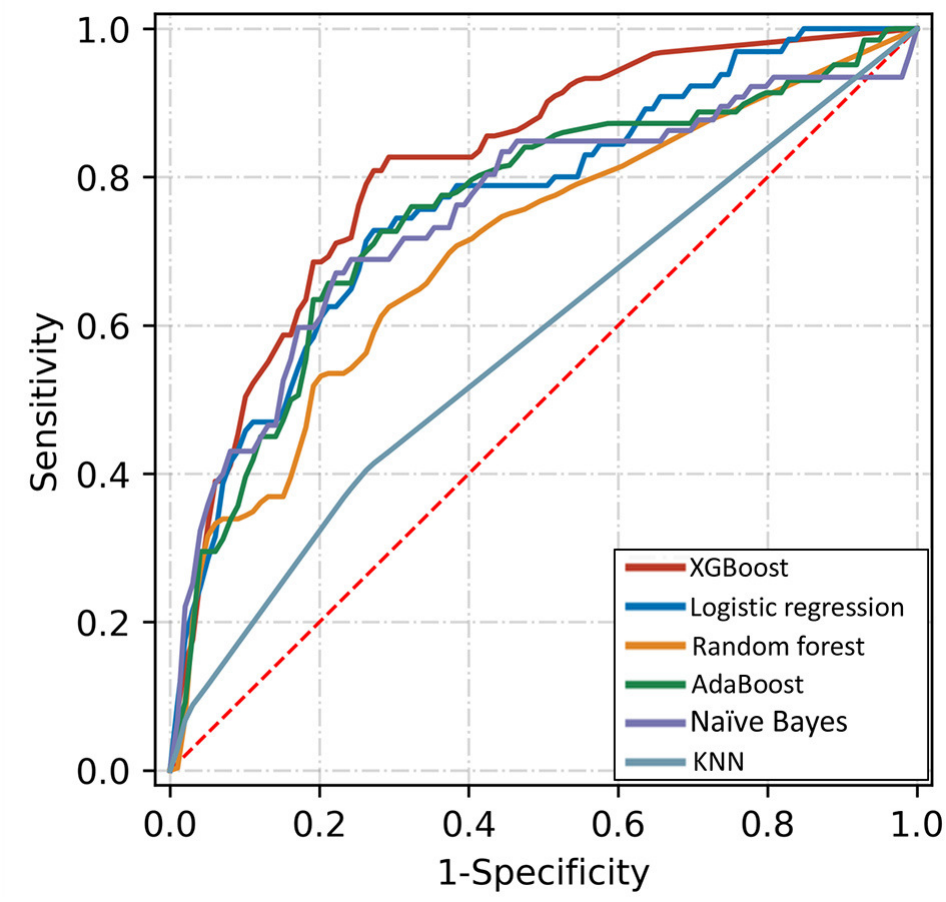
Receiver operating characteristic curves of five algorithms for detecting referable diabetic retinopathy based on top-10 important variables.

Table 3 shows the discriminative performance of the algorithms using five-fold crossvalidation and an 80:20 ratio of training and validation. Figure 4 shows the performance of top-5 models. The XGBoost algorithm was nominally the best with an AUC of 0.816 (95%CI: 0.812, 0.820). The AUCs for logistic regression, AdaBoost, naïve Bayes, and Random forest were 0.766 (95%CI: 0.756, 0.776), 0.754 (95%CI: 0.744, 0.764), 0.753 (95%CI: 0.743, 0.763), and 0.705 (95%CI: 0.697, 0.713), respectively.

**Table 3:** The performance of machine learning models for diagnosing referable diabetic retinopathy.

	**AUC**	**Accuracy**	**Sensitivity**	**Specificity**	**PPV**	**NPV**
XGBoost	0.816 (0.033)	0.796 (0.064)	0.796 (0.132)	0.799 (0.073)	0.179 (0.039)	0.981 (0.007)
Logistic regression	0.766 (0.083)	0.797 (0.054)	0.683 (0.159)	0.808 (0.062)	0.174 (0.053)	0.972 (0.008)
AdaBoost	0.754 (0.087)	0.755 (0.044)	0.743 (0.114)	0.761 (0.046)	0.137 (0.035)	0.974 (0.010)
Naïve Bayes	0.753 (0.090)	0.788 (0.037)	0.689 (0.126)	0.799 (0.033)	0.159 (0.049)	0.972 (0.010)
Random forest	0.705 (0.070)	0.776 (0.080)	0.622 (0.204)	0.768 (0.106)	0.151 (0.058)	0.965 (0.009)
Light GBM	0.640 (0.098)	0.941 (0.012)	0.358 (0.249)	0.901 (0.084)	-	0.956 (0.012)
KNN	0.577 (0.048)	0.930 (0.025)	0.316 (0.197)	0.839 (0.116)	-	0.946 (0.016)
ANN	0.475 (0.041)	0.584 (0.215)	0.570 (0.241)	0.588 (0.242)	0.054 (0.012)	0.958 (0.015)

*AUC, area under ROC curve; PPV, positive predictive value; NPV, negative predictive value; XGBoost, extreme gradient boosting; KNN, k-nearest neighbor; ANN, artificial neural network; AdaBoost, adaptive boosting; GBM, gradient boosting machine*.

## Discussion

This study developed and validated an ML-based model for screening RDR in a Chinese population using common and readily available variables. After ranking the importance of the risk factors, the top 10 essential risk factors were adopted for modeling by eight ML models. The XGBoost classifier exhibited the best performance with an AUC of 0.816, which was validated in an independent population. To our knowledge, this is the first diagnostic model for RDR in the Chinese diabetic population based on ML and simple variables, which has the potential for accurate and rapid RDR screening.

State-of-the-art ML methods were adopted in this study. Traditional regression analysis relies on hypothesis-driven assumptions, while the ML techniques used do not require a predetermined assumption. This feature allows for data-driven exploration for non-linear patterns that predict risk for a given individual, i.e., precise risk stratification (10, 19). As observed in this study, the ranking of the importance showed that the duration of diabetes, HbA1c, systolic blood pressure, TG, BMI, serum creatinine, age, education level, duration of hypertension, and income level were the 10 most important factors for RDR. Furthermore, the given ML algorithm requires only minimal input during the model development stage, which is particularly important given that ML models can easily incorporate new data to update and optimize, thereby continuously improving their discriminative performance over time (20). Our models provided information for DR screening in high-risk populations and can help to reduce the frequency of ocular examinations in low-risk populations (21).

Limited studies were available on risk stratification of DR based on ML and non-ocular parameters. Azizi-Soleiman et al. reported a model for detecting DR in Iranians based on outpatient clinical data (22). By training the data of 1,782 patients (without using cross-validation), the logit model obtained an AUC of 0.760 based on backward elimination as a feature selection strategy (22). Tsao et al. divided the clinical data of 536 patients in Taiwan into training and validation sets (at an 80:20 ratio), and compared the performance of four models (support vector machine, decision tree, ANN, and logistic regression) for DR detection, and found that support vector machine performed best with an AUC of 0.839 (14). Yao et al. reported that a back propagation artificial neural network outperformed logistic regression for DR detection with AUCs of 0.84 and 0.77, respectively (13). The abovementioned studies were based on hospital-based data, but population-based data are more relevant to the reality of DR screening programmes (5). This study applied ML techniques to population-based data and demonstrated their usefulness for RDR detection with similar AUCs to those in hospital-based studies.

The XGBoost algorithm, which has attracted attention in recent years due to its excellent performance and efficient training speed, performed best in this study. This model has been evaluated in several other ocular diseases. Oh et al. compared four ML models (support vector machine, C5.0, random forest, and XGboost) for detecting glaucoma and reported that XGboost performed best with an AUC of 0.945, accuracy of 0.947, sensitivity of 0.941, and specificity of 0.950 (23). Xu et al. demonstrated that the XGBoost classifier had the highest accuracies for predicting subretinal fluid absorption at 1, 3, and 6 months in patients with central serous chorioretinopathy (24). Wu et al. reported that the intraocular pressure in children with myopia treated with topical atropine can be predicted by using ML methods, and the XGBoost ranks the best predictive models (25). The present study confirmed that XGBoost is also a good tool for DR screening.

This study has several strengths. First, all variables were derived from easily accessible non-ocular examinations and questionnaires. The model is especially suitable for primary hospitals and diabetic clinics without the need for expensive laboratory tests and ocular specialists equipped with ophthalmic equipment, which is especially useful in areas of low socio-economic status and with limited health resources. Second, the model is derived from a large population-based survey in China, making it highly representative and generalisable. Third, the majority of previous studies divided smoking and alcohol consumption into only two categories (with or without), and therefore they do not reflect the effect of frequency and quantity on disease. The importance ranking analysis showed that the amount and duration of smoking and drinking were also important for RDR. Finally, the ranking of risk factors might provide insight into the prevention of DR. This study also has limitations. Only Chinese adults were included in the present study; however, ethnic variations in DR onset and progression have been confirmed in population studies (26, 27). Therefore, this study needs to be repeated with other races. In addition, this study evaluated the feasibility and performance of ML, but not its implementation. However, a population-based study is especially suited to assessing the initial feasibility of ML algorithms in the real world.

## Conclusion

In this secondary analysis of a large-scale population-based survey, we first extracted demographic variables, laboratory test results, and medical and family history, and then applied different ML algorithms to rank risk factors and for identification of RDR. The XGBoost algorithm achieved the best performance based on 10 simple variables. The usage of ML algorithms to rank epidemic risk factors (other than ophthalmic examinations) to identify referable patients will reduce the cost and had a high application valuable in resource-poor areas in China.

## Data Availability Statement

Data are available from the authors upon reasonable request and with permission of Guangdong Provincial People's Hospital.

## Ethics Statement

Written informed consent was obtained from the individual(s) for the publication of any potentially identifiable images or data included in this article.

## Author Contributions

CY and QL: conceptualization, investigation, formal analysis, and writing—original draft. HG: validation, resources, material support, administrative, and writing—review and editing. MZ: investigation, resources, material support, and administrative. LZ: investigation, material support, and review and editing. GZ: data analysis and review and editing. QM: project administration, conceptualization, investigation, supervision, formal analysis, and writing—review and editing. YC: project administration, conceptualization, investigation, supervision, formal analysis, and writing—review and editing. All authors contributed to the article and approved the submitted version.

## Funding

This research was supported by the National Natural Science Foundation of China, Beijing, China (81800829, 82000897); Guangdong Basic and Applied Basic Research Foundation (2019A1515010697); the Guangzhou Science and Technology Program Project (202002030400). The funding organizations had no role in the design or conduct of this research.

## Conflict of Interest

The authors declare that the research was conducted in the absence of any commercial or financial relationships that could be construed as a potential conflict of interest.

## Publisher's Note

All claims expressed in this article are solely those of the authors and do not necessarily represent those of their affiliated organizations, or those of the publisher, the editors and the reviewers. Any product that may be evaluated in this article, or claim that may be made by its manufacturer, is not guaranteed or endorsed by the publisher.
