# Supplementary material for: Usefulness of Machine Learning for Identification of Referable Diabetic Retinopathy in a Large-Scale Population-Based Study
Source: Front Med (Lausanne). 2021 Dec 9;8:773881. doi: 10.3389/fmed.2021.773881 (PMC8717406; doi:10.3389/fmed.2021.773881)
Supplement: Supplementary file 1 [file Table_4.docx]

**Supplementary table.** Top 20 importance factors in each machine learning model for diagnosing referable diabetic retinopathy.

| **Rank** | **XGBoost** | | **Random forest** | | **Naïve Bayes** | | **KNN** | |
| --- | --- | --- | --- | --- | --- | --- | --- | --- |
|  | **Variable** | **Gain** | **Variable** | **Importance** | **Variable** | **Importance** | **Variable** | **Importance** |
| 1 | DM duration | 331.854 | DM duration | 0.103 | DM duration | 0.225 | SBP | 0.131 |
| 2 | FPG | 162.703 | FPG | 0.085 | Alcohol drinking habit | 0.208 | Serum creatine | 0.130 |
| 3 | BUN | 154.797 | BUN | 0.077 | History of HL | 0.065 | DBP | 0.115 |
| 4 | Body mass index | 139.249 | HbA1c | 0.066 | Use of insulin | 0.057 | Age | 0.110 |
| 5 | Total cholesterol | 137.658 | Triglyceride | 0.065 | Education level | 0.052 | DM duration | 0.090 |
| 6 | Triglyceride | 133.159 | Total cholesterol | 0.065 | Alcohol consumption | 0.046 | Years of smoking | 0.071 |
| 7 | HbA1c | 128.818 | Serum creatine | 0.061 | Marital status | 0.034 | Body mass index | 0.067 |
| 8 | SBP | 120.389 | Age | 0.056 | Triglyceride | 0.032 | FPG | 0.060 |
| 9 | Waist to hip ratio | 88.669 | SBP | 0.056 | Years of drinking | 0.025 | Years of hypertension | 0.059 |
| 10 | Serum creatine | 85.251 | Body mass index | 0.052 | Years of smoking | 0.022 | HbA1c | 0.028 |
| 11 | Age | 75.054 | Waist to hip ratio | 0.049 | Years of hyperlipidemia | 0.021 | BUN | 0.024 |
| 12 | DBP | 70.933 | DBP | 0.040 | Family history of DM | 0.021 | No. of child | 0.020 |
| 13 | Years of smoking | 46.442 | No. of child | 0.028 | Smoking status | 0.019 | Years of drinking | 0.016 |
| 14 | Occupation | 43.592 | Years of hypertension | 0.027 | Waist to hip ratio | 0.019 | Total cholesterol | 0.015 |
| 15 | No. of child | 23.630 | Education level | 0.021 | Years of hypertension | 0.017 | Triglyceride | 0.015 |
| 16 | Years of hypertension | 21.178 | Years of smoking | 0.021 | Sex | 0.016 | Years of hyperlipidemia | 0.014 |
| 17 | Education level | 13.101 | Level of family income | 0.018 | Serum creatine | 0.013 | Smoking amount | 0.012 |
| 18 | Family history of DM | 9.090 | Occupation | 0.012 | FPG | 0.012 | Alcohol consumption | 0.010 |
| 19 | Level of family income | 8.105 | Years of hyperlipidemia | 0.011 | SBP | 0.012 | Level of family income | 0.008 |
| 20 | Smoking status | 7.668 | Smoking amount | 0.011 | Age | 0.011 | Education level | 0.007 |

FPG= Fasting plasma glucose; BUN= Blood urea nitrogen; History of HL=History of hyperlipidemia.
